# Synthesis and magnetic transitions of rare-earth-free Fe–Mn–Ni–Si-based compositionally complex alloys at bulk and nanoscale

**DOI:** 10.3762/bjnano.16.62

**Published:** 2025-06-05

**Authors:** Shabbir Tahir, Tatiana Smoliarova, Carlos Doñate-Buendía, Michael Farle, Natalia Shkodich, Bilal Gökce

**Affiliations:** 1 Chair of Materials Science and Additive Manufacturing, University of Wuppertal, Gaußstr. 20, 42119 Wuppertal, Germany; 2 Faculty of Physics and Center for Nanointegration Duisburg-Essen (CENIDE), University of Duisburg-Essen, Lotharstr. 1, 47057 Duisburg, Germany; 3 GROC·UJI, Institute of New Imaging Technologies, Universitat Jaume I, Av. De Vicent Sos Baynat s/n, 12071 Castellón, Spain

**Keywords:** compositionally complex alloys, magnetic phase transition, nanoparticles, pulsed laser ablation in liquid, rare-earth free

## Abstract

Magnetic phase transitions at the Curie temperature are essential for applications like magnetocaloric refrigeration, magnetic sensors, and actuators, but the reliance on costly, scarce rare-earth materials limits sustainability. Developing affordable, rare-earth-free materials with tunable magnetic properties and scalable miniaturization methods is vital for advancing technology. We present a comprehensive synthesis approach for rare-earth-free compositionally complex alloys (CCAs) with magnetic phase transitions, spanning from bulk materials to nanoparticles. Specifically, we investigate Mn_22.3_Fe_22.2_Ni_22.2_Ge_16.65_Si_16.65_ (Ge-based CCA) and Mn_0.5_Fe_0.5_NiSi_0.93_Al_0.07_ (Al-based CCA). The bulk materials are prepared by ball milling and spark plasma sintering or powder pressing and sintering. Nanoparticles (NPs) from the bulk materials are synthesized by pulsed laser ablation in liquid. Magnetization measurements confirm a ferromagnetic-to-paramagnetic phase transition in bulk alloys, with *T*_c_ = 179 K for Ge-based CCA and *T*_c_ = 263 K for Al-based CCA. At the nanoscale, both Ge- and Al-based NPs exhibit superparamagnetic behaviour, with blocking temperatures of *T*_B_ ≈ 120 K for Ge-based NPs (*x*_c_ = 13.4 ± 15.5 nm, average particle size) and *T*_B_ ≈ 100 K for Al-based NPs (*x*_c_ = 18.4 ± 9.1 nm, average particle size), demonstrating the intrinsic superparamagnetic nature of NPs. While the Ge-based CCA demonstrates almost twice the saturation magnetization (*M*_s_) and ≈20% lower hysteresis (*H*_c_) in bulk form, the Al-based CCA exhibits comparable *M*_s_ and ≈45% lower *H*_c_ at the nanoscale at 5 K. These results indicate that the Al-based CCA is a promising, cost-effective alternative to Ge-based CCA at nanoscale, providing an economically viable and cost-effective alternative for nanoscale-based applications.

## Introduction

Magnetic phase transitions are characterized by changes in the material’s magnetic properties in response to varying conditions such as applied magnetic or electric fields, temperature, and/or pressure. In particular, the magnetic phase transition at the Curie temperature (*T*_c_) is a type of magnetic phase transition characterized by the loss of spontaneous magnetization in ferromagnetic materials [1]. One key application of such transitions is in magnetocaloric cooling systems where magnetocaloric materials, when cycled near *T*_c_ in an external magnetic field, exhibit an adiabatic temperature change, enabling energy-efficient and environmentally friendly refrigeration [2]. These solid-state cooling systems are being developed as alternatives to conventional gas-based refrigeration and are especially advantageous for applications requiring tailored temperature ranges, such as room-temperature cooling and cryogenic systems [3–4]. The magnetic transition also plays a critical role in the development of temperature-sensitive magnetic sensors and actuators [5–6]. These devices harness the abrupt change in magnetic properties at *T*_c_ to detect temperature fluctuations or trigger mechanical responses, making them essential in automation, industrial processes, and healthcare monitoring technologies.

Considerable research has been dedicated to tailoring the Curie temperature and associated magnetic properties through material design. Advances in composition modification, doping strategies, and material synthesis have been shown to effectively tune the phase transition characteristics, such as the temperature, coercivity (*H*_c_), magnetization, and Curie or Néel temperatures [7–9]. For instance Zhou et al. [10] reported that adjusting the composition of NiMnGa to Ni_55.2_Mn_18.6_Ga_26.2_, a giant magnetocaloric response with a Δ*S* of −20.4 J·kg^−1^·K^−1^ at 317 K in a 5 T field can be achieved compared to Ni_57.2_Mn_15.9_Ga_27.0_ where a Δ*S* of just −2 J·kg^−1^·K^−1^ at 310 K was witnessed.

Within the myriad of material systems exhibiting magnetic transitions, compositionally complex alloys (CCAs) have garnered considerable attention because of their compositional flexibility and exceptional thermomechanical [11–12], magnetic [13–14], and electrical insulation properties [15–16]. CCAs are composed of five or more elements, offering design freedom. This flexibility allows for elemental combinations that control the configurational entropy of mixing, phase, and free energy of the material. Consequently, the magnetic phase transition of CCAs can be tuned by altering their chemical composition because of the different elemental interactions [17]. A significant advantage of using CCAs is their potential to replace rare earth elements in magnetic materials [18]. The development of rare-earth-free or rare-earth-lean magnets is critical because of the economic, environmental, and supply chain challenges associated with rare earth elements [19–20]. For instance, mining and processing of rare earths are linked to significant environmental risks, while geopolitical factors pose supply chain vulnerabilities [21]. By avoiding the use of rare earth elements, CCAs present a sustainable alternative for functional magnetic materials [14]. Specifically, MnTX-based CCA alloys (where T is the transition metal and X can be Si, Ge or Al) have gained attention because of their magneto-structural phase transition at low temperatures. As ternary alloys, these materials undergo a magnetic and structural transition from a low-temperature orthorhombic TiNiSi-type structure to a high-temperature hexagonal Ni_2_In-type structure [22]. For instance, a ternary MnNiSi alloy transitions from the hexagonal Ni_2_In structure to the orthorhombic TiNiSi structure at 1200 K [23], which is far from ideal for magnetocaloric, electronic, and spintronic-based applications. When these alloys are doped with elements such as Fe (which partly substitutes Mn atoms) and Ge or Al (which partially replaces Si atoms) [24] (forming CCAs), it effectively lowers the structural and magnetic phase transition temperatures while maintaining the overall magnetization. Previous studies on bulk MnFeNiGeSi [25] (i.e., doping MnNiSi with Fe and Ge) and MnFeNiSiAl [24] (i.e., doping NiMnSi with Fe and Al) alloys, synthesized by arc melting of pure elements show a second-order magnetostructural phase transition between 170 and 220 K with an isothermal entropy change of −7.3 J·kg^−1^·K^−1^ at 2.5 T and a first-order magnetostructural phase transition near 200 K with an isothermal entropy change of −23 J·kg^−1^·K^−1^ at 2 T, respectively.

Bulk CCAs are explored thanks to the metallurgy approaches that allow for material composition control and alloying. However, it is desirable to achieve controlled methodologies to downsize CCAs to the nanoscale because they exhibit remarkable properties due to the interplay between their compositional complexity and nanoscale effects, such as a high surface-to-volume ratio and quantum confinement. These unique characteristics make CCA nanoparticles (NPs) highly suitable for catalysis [26–27], energy storage [28], wear resistant coatings [29], environmental [30], biomedical [31], magnetic [32] and electronic [33] technologies. In magnetic NPs, a key feature is the superparamagnetic blocked-to-superparamagnetic fluctuating transition, which occurs at a characteristic blocking temperature (*T*_B_). Below *T*_B_, NPs exhibit superparamagnetic behavior, where magnetic moments of particles fluctuate due to thermal energy but can be aligned under an external field. Above *T*_B_, thermal fluctuations dominate, causing a transition to a superparamagnetic fluctuating state. This transition is particularly relevant for applications such as magnetic hyperthermia, where NPs are used in cancer therapy to induce localized heating when exposed to an alternating magnetic field [34].

However, producing CCAs at the nanoscale presents significant challenges. Traditional wet chemistry approaches often fail because of elemental immiscibility under equilibrium conditions, which leads to elemental segregation and phase separation [35]. Additionally, standard near-equilibrium heating methods are not suitable for synthesizing CCA NPs because of inherent thermodynamic limitations [36]. While high-temperature synthesis techniques have been successful in producing CCA NPs, they come with certain drawbacks. For instance, carbothermal synthesis requires an electrically conductive substrate, making it unsuitable for large-scale production [37]. Pyrolysis requires purification steps to eliminate polymer impurities and also result in phase segregation due to differences in precursor reduction temperatures [38]. Other techniques such as laser scanning ablation [39] and Joule heating [40] have also been employed to generate CCAs, but they often involve costly precursors and lengthy solvent screening processes.

Among the various techniques available for producing CCA NPs, pulsed laser ablation in liquids (PLAL) stands out as a particularly promising method [41–43]. PLAL is a straightforward and versatile method that does not require expensive precursors, reducing agents, or surfactants [44–45]. The process is based on the laser irradiation of the target material submerged in a liquid environment. This makes PLAL a safe, scalable and environmentally friendly approach [46–48]. Research on the synthesis of CoCrFeMnNi Cantor alloy NPs by PLAL demonstrated that this method consistently produces NPs with near-equiatomic compositions, regardless of the target preparation technique. Additionally Gatsa et al. [49] provide a realistic perspective that the CCA NPs production using a multibeam PLAL approach can be scaled up to ca. 3 g·h^−1^. The CCA NPs produced by PLAL have shown a promising response as catalysts for oxygen reduction reactions [50].

In the current work, we aim to develop NPs of bulk rare-earth-free MnTX-based CCAs via PLAL. We start with bulk Ge-based CCA synthesized by high energy ball milling (HEBM) followed by spark plasma sintering (SPS), and an Al-based CCA obtained by powder pressing and sintering. Our goal is not only to replace rare earth elements but also to substitute other expensive elements, such as Ge, with more cost-effective alternatives such as Al. Notably, Ge is nearly a thousand times more expensive than Al, making this substitution economically advantageous [51]. By employing a more efficient and reliable alloy production method, we aim to create sustainable magnetic materials. Additionally, we compare the composition and magnetic transitions of the CCAs at the nanoscale to assess how these factors influence material magnetic performance. This research addresses the economic challenges associated with costly elements and rare earth materials, providing a pathway for developing bulk and nanometric functional magnetic materials that meet the demands of modern technological applications.

## Materials and Methods

### Preparation of bulk Ge-based CCAs

**Figure 1:**
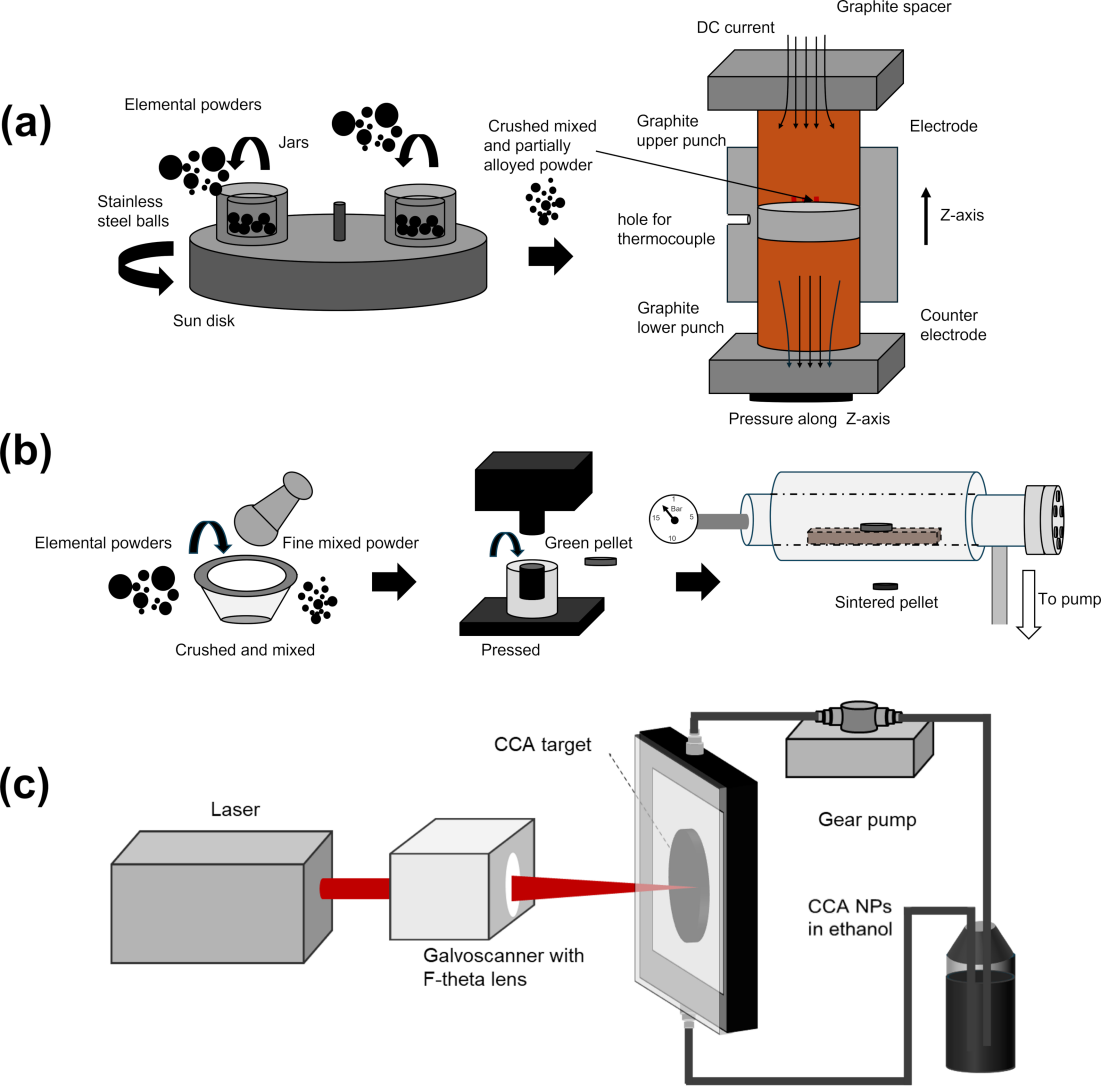
Synthesis route of the Ge- and Al-based CCAs. (a) Bulk Ge-based CCA produced via HEBM of elemental powders followed by SPS. (b) Bulk Al-based CCA synthesized through a process of mixing elemental powders using mortar and pestle, followed by uniaxial pressing and vacuum oven sintering. (c) CCA NPs produced by picosecond-pulsed laser ablation of the CCA alloy targets in ethanol employing a closed loop liquid flow chamber.

The bulk Ge-based CCA was synthesized by a two-step process. Initially, elemental Mn (99.2%, 3 µm), Fe (99.96%, 10–20 µm), Ni (99.5%, 45–60 µm), Si (99.999%, 45–60 µm), and Ge (99.99%, <250 µm) powders were mixed in the desired stoichiometric proportions and underwent HEBM in a planetary ball mill for 90 min under argon atmosphere and a ball-to-powder weight ratio of 20:1 with a rotation speed of sun disk/jars 700 rpm/1400 rpm. In the second step, the HEBM powders were consolidated using SPS (Dr. Sinter Lab – Fuji Electronic Industrial Co. Ltd.) in a vacuum environment. The powder mixture was loaded into a cylindrical graphite die with an inner diameter = 10 mm and uniaxially compressed at 50 MPa. SPS was carried out at 1073 K with a dwelling time of 10 min and a heating rate of 100 K·min^−1^. The temperature was measured by a K-type thermocouple placed in a radial hole inside the die. The SPS-processed disks had a thickness of *d* = 3–4 mm and a diameter of ∅ = 10 mm. The preparation procedure for the bulk Ge-based CCA is illustrated in Figure 1a.

### Preparation of bulk Al-based CCAs

The bulk Al-based CCA was produced from elemental Mn (99.6%, <10 µm), Fe (99.5%, 6–10 µm), Ni (99.9%, 3–7 µm), Al (99.5%, 44 µm), and Si (99%, 44 µm) powders. The bulk targets preparation process is illustrated in Figure 1b. To achieve the desired composition, each elemental powder was weighed and mixed in the correct proportions by mass. The powders were thoroughly blended for 20 min using a mortar and pestle, as shown in the left panel of Figure 1b. After mixing, the powder was compressed at 100 MPa to produce cylindrical pellets of 10 mm in diameter and 2 mm in thickness (middle panel, Figure 1b). Finally, the samples underwent heat treatment in a near-vacuum environment (down to 10 mbar) at 600 °C for seven days, followed by rapid quenching in water. This procedure helps achieving a homogeneous alloy and is a cost-effective and time-efficient alternative to conventional target preparation methods, such as arc melting or HEBM followed by SPS, and suitable for CCA NP generation because of the inherent alloying produced during PLAL processing.

The bulk CCAs were ground, polished, and analyzed by SEM (JEOL JSM-7600 F, Japan). The chemical composition was determined using energy-dispersive X-ray spectroscopy (EDX) with an Oxford Inca spectrometer.

The crystal structure of bulk CCAs was characterized by X-ray diffraction (XRD) using a DRON-4–07 diffractometer with Co Kα radiation over a 2θ range of 20–110°. The magnetic properties of the samples were measured using a Quantum Design DynaCool physical property measurement system at temperatures ranging from 5 to 390 K under external magnetic fields up to 9 T.

### Synthesis of CCA NPs by PLAL

The CCA colloidal NPs were synthesized by PLAL using the bulk Ge-based and Al-based CCA targets submerged in ethanol (Figure 1c). A near-infrared picosecond-pulsed Nd:YAG laser source (Coherent, HyperRapid NX, Kaiserslautern, Germany, 10 ps, 1064 nm) was employed to irradiate the targets within ethanol at a laser fluence of 2.8 J·cm^−2^ [41]. Additionally, to increase the NP concentration and reduce the organic solvent use, a closed loop liquid flow system was employed.

Size distribution, morphology, elemental composition, and crystal structure of the NPs from both CCA targets were examined using transmission electron microscopy (TEM) and EDX with a Jeol 2200FS microscope (Japan) equipped with an Oxford X-MaxN TLE 80 EDX detector (UK). The microscope was operated at an acceleration voltage of 200 kV and utilized a 2k × 2k GATAN UltraScan 1000XP CCD camera. For TEM analysis, the colloidal particles were dispersed onto a carbon-supported TEM copper grid and dried under ambient conditions. The particle size distribution was determined by measuring the Feret diameter of individual particles from TEM images using ImageJ software [52]. The NP crystal structure was evaluated using CrysTBox software [53] using digital diffractograms. EDX data was processed using AZtec software.

## Results and Discussion

### Microstructural characterization of bulk CCAs

**Figure 2:**
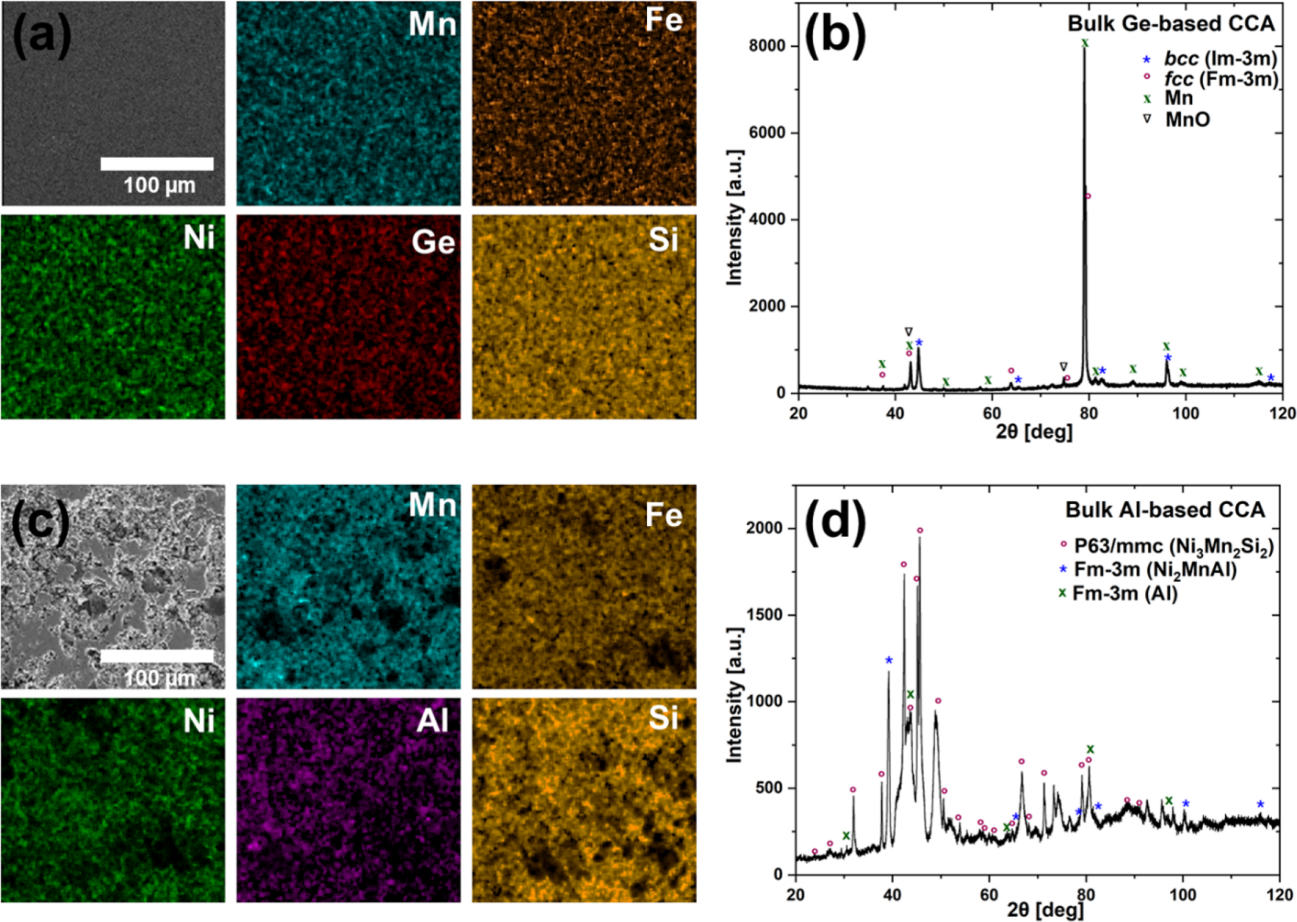
(a, c) SEM (SE) and EDX elemental maps of Mn, Fe, Ni, Ge, and Si obtained for bulk the Ge-based CCA and Mn, Fe, Ni, Al, and Si, obtained for bulk Ge-based CCA. (b, d) X-ray diffraction (XRD) patterns of the bulk Ge-based CCA and the bulk Al-based CCA, showing the presence of side phases.

The SEM images and EDX elemental maps of the polished surface of the Ge-based CCA (Figure 2a) revealed a homogeneous microstructure, with no significant elemental segregation on the micrometer scale, indicating a successful mixing and compaction. This uniformity results from the HEBM, which facilitates pre-alloying of elemental powders, followed by SPS. The controlled heating during SPS helps to prevent the grain growth and retains the nanoscale structure from HEBM powders. Table 1 presents the average composition of the Ge-based and Al-based CCAs measured at different spatial positions of the target’s surface, showing that the measured values nearly align with the expected alloy composition. Notably, the percentage variance in Table 1 highlights that Ge exhibits the highest variance (14.1%) compared to other elements. This may be attributed to higher diffusion rates due to its lower latent heat of fusion (31.8 kJ·mol^−1^) and its relatively low melting point (1211 K) compared to other constituents, as observed by Tiwari and colleagues [54]. Additionally, the target composition was re-evaluated from both the surface and cross-section, confirming that the composition remains consistent throughout the surface and bulk of the CCA within the limits of experimental error (Figure S1 and Table S1, Supporting Information File 1). Figure 2b provides an overview of the alloy’s crystallographic information, indicating the coexistence of BCC and FCC phases. This differs from the work by Law et al. [25] where the alloy synthesized by arc melting resulted in a single-phase HCP structure at room temperature. The BCC phase, predominant in the current work, may not appear in alloys processed by arc melting due to the higher cooling rates (≈2000 K·s^−1^) [55], which can stabilize HCP structures through a quenching effect [56] that does not occur for the SPS cooling rates (1.6 to 6.9 K·s^−1^) [57]. The appearance of the FCC phase is consistent with Mn segregation and possible MnO formation, as evidenced by XRD peaks. The microsegregation of Mn can be due to a phenomenon consistent with observations in other CCAs, often attributed to the elastic strain energy [58] or due to the presence of oxygen on the surface forming MnO [59].

**Table 1:** Comparison of the surface composition with the expected bulk composition, along with the percentage variance for both bulk Ge-based and Al-based CCAs.

Bulk Ge-based CCA				Bulk Al-based CCA			

	expected bulk composition (*E*) [atom %]	surface composition (*S*) [atom %]	percent variance = 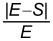 × 100 [%]		expected bulk composition [atom %]	surface composition [atom %]	percent variance = 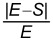 × 100 [%]

Mn	22.3	22.7 ± 0.9	1.8	Mn	16.67	20.8 ± 8.1	24.8
Fe	22.2	24.3 ± 0.7	9.5	Fe	16.67	17.5 ± 2.9	5.0
Ni	22.2	20.8 ± 0.8	6.3	Ni	33.3	31.2 ± 2.8	6.3
Ge	16.65	14.3 ± 0.5	14.1	Al	2.3	5.10 ± 0.6	121.7
Si	16.65	17.9 ± 1.8	7.5	Si	31.0	25.4 ± 2.3	18.1

As expected, the SEM images and EDX elemental mapping of the polished Al-based CCA surface (Figure 2b) show a more porous structure and inhomogeneous elemental distribution, with noticeable segregation. This results from a less effective mixing during the powder mixing process in the mortar, compared to the Ge-based CCA prepared by HEBM with subsequent consolidation by SPS. The lower sintering temperature and absence of pre-alloying limits the diffusion of the elements, leading to incomplete alloying. The composition exhibits a much larger percentage variance from the expected bulk composition compared to the Ge-based CCA. Al with its much lower latent heat of fusion (10.7 kJ·mol^−1^) and melting point (933.5 K) compared to Ge, segregates easier, resulting in a significantly higher percentage variance (121.7%) for Al compared to other elements. However, it is important to note that the percentage variance is strongly influenced by the absolute elemental concentration; thus, even small absolute deviations at low concentrations can result in disproportionately high percentage variance. XRD analysis revealed the formation of multiphase alloys, with identifiable peaks corresponding to Al-deficient and Al-rich phases. The Al-deficient phase, Ni_2_Mn_2_Si, was found to have a hexagonal (*P*6_3_/*mmc*) structure, while a cubic (*Fm*−3*m*) structure was observed in Ni_2_MnAl and elemental Al. The hexagonal structure was also found in the Al-based CCA produced by Biswas and colleagues [24]. The elemental characterization by SEM/EDX from different surface positions (see Figure S2 and Table S2, Supporting Information File 1), further supports the formation of multielement phases. Nevertheless, the purpose of the produced target through this procedure is not to directly generate a bulk CCA, but to prepare in a fast, up-scalable, and economically feasible manner intermixed targets with the CCA components that can be later in-situ alloyed during PLAL to generate compositionally controlled CCA NPs.

### CCA NP synthesis and characterization

#### Particle size distribution

**Figure 3:**
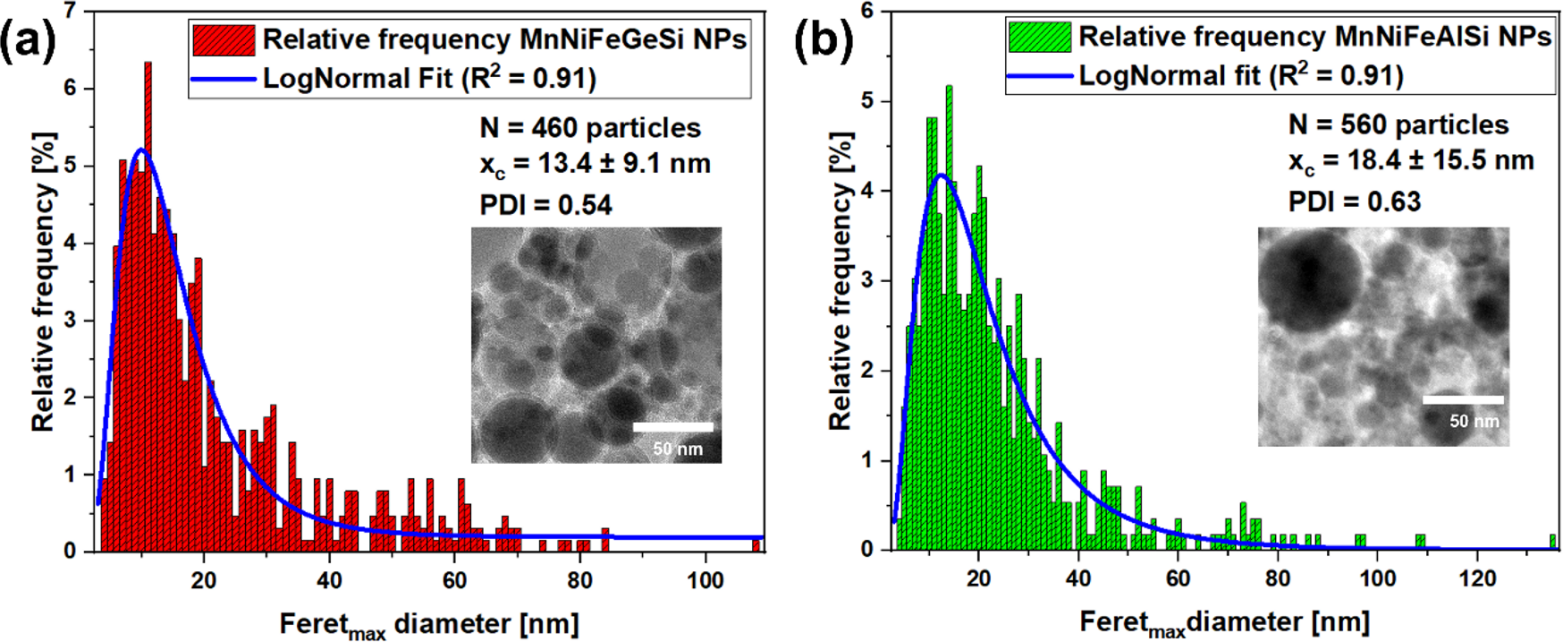
Particle size distribution extracted from TEM images (inset) from (a) bulk Ge-based CCA and (b) bulk Al-based CCA target.

The NPs synthesized by pulsed laser ablation in ethanol exhibit a log–normal particle size distribution ranging from 2 to 130 nm for both Ge- and Al-based CCAs (Figure 3). The polydispersity index (PDI) exceeds 0.3, indicating a polydisperse size distribution. This polydispersity is further supported by simulation studies by Shih et al. [60], where small NPs are formed following the phase explosion process, in which a superheated region of the target decomposes into vapor, small clusters, and droplets, and large particles result from photomechanical spallation, leading to the ejection of larger droplets. The average particle size of the Al alloy NPs (*x*_c_ = 18.4 ± 15.5 nm) is larger than that of Ge-based CCA NPs (*x*_c_ = 13.4 ± 9.1 nm). This difference can be attributed to variations in alloy composition and material properties, which influence the ablation plume dynamics and particle formation kinetics during PLAL. Specifically, the thermal properties, such as melting point and heat conductivity, and the volatility of the alloy components affect the balance between phase explosion and spallation mechanisms, ultimately leading to differences in particle size distribution.

#### Composition analysis

**Figure 4:**
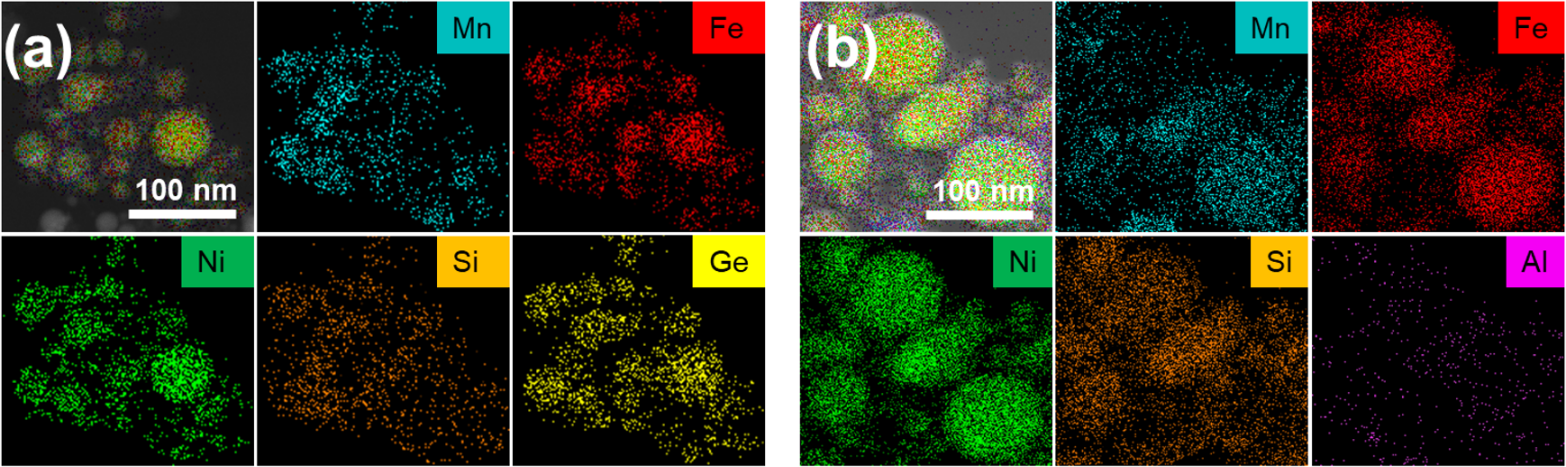
(a) Mn, Fe, Ni, Ge, and Si EDX elemental maps of the PLAL-generated CCA NPs from the bulk Ge-based CCA target. (b) Mn, Fe, Ni, Al, and Si EDX elemental maps of the PLAL generated CCA NPs from the Al-based CCA target.

The EDX mapping of Ge-based CCA NPs shows that the particles contain all constituent elements of the target (Figure 4a). As shown in Table 2, the percentage variation of each element in the CCA NPs is higher than in the bulk alloy when compared to the expected composition, and the standard deviation between NPs is also greater than that of the bulk alloys.

**Table 2:** Comparison of the NP composition with the expected bulk composition, along with the percentage variance for both Ge-based and Al-based CCAs.

Ge-based CCA NPs				Al-based CCA NPs			

	expected NP composition (*E*) [atom %]	average NP composition (*N*) [atom %]	percent variance = 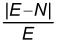 × 100 [%]		expected NP composition (*E*) [atom %]	average NP composition (*N*) [atom %]	percent variance = 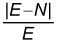 × 100 [%]

Mn	22.3	15.3 ± 4.6	31.4	Mn	16.67	12.0 ± 1.8	28.0
Fe	22.2	21.6 ± 3.9	2.7	Fe	16.67	18.3 ± 0.3	9.8
Ni	22.2	25.4 ± 4.1	14.4	Ni	33.3	35.3 ± 4.1	6.0
Ge	16.65	19.8 ± 1.9	18.9	Al	2.3	1.30 ± 0.4	43.5
Si	16.65	17.8 ± 2.1	6.9	Si	31	33.0 ± 5.0	6.5

This discrepancy is due to the different synthesis mechanisms of NPs and bulk alloys and the intrinsic difficulties associated with the synthesis of CCA NPs. Beyond the elemental composition of the bulk target, the PLAL NP composition depends on the volatility and ionization potential of each element. Hence, the laser ablation of multicomponent targets has an impact on NP stoichiometry, since the more volatile components evaporate more efficiently. The greatest deviation was observed for Mn, with a variance of 31.4% below the expected composition, compared to just 1.8% variance in the bulk Ge-based CCA. This was also observed in our previous work [41] on Cantor alloy NPs synthesized via PLAL, where separate Mn-rich clusters formed due to elemental evaporation during synthesis. Additionally, the ionization potential influences the ablation plume dynamics. Notably, the Ge variation from the expected composition is lower than for Mn, contrasting with the bulk alloy’s surface composition. This can be attributed to Mn’s lower ionization potential (7.34 eV) compared to Ge (7.90 eV). Furthermore, HRTEM imaging (Figure S3a, Supporting Information File 1) reveals the formation of a ≈2.5 nm thick oxide shell surrounding the NPs. A line scan analysis (Figure S3b, Supporting Information File 1) confirms an elevated oxygen concentration at the NP surface, indicating surface oxidation effects. Most oxide shells, such as MnO, NiO, or FeO are antiferromagnetic [61] and exhibit higher anisotropy than the NP core, leading to an exchange bias effect in the hysteresis loop and an increase in coercivity [62].

EDX mapping of the Al-based CCA NPs (Figure 4b) confirms that all constituent elements from the target material are present within the particles. Table 2 shows the NP composition deviation from the bulk target. The largest percentage variation in composition was found to be in Al content, which is 43.5% lower than the expected composition. This can also be due to the lower melting point of Al and lower ionization potential of 5.99 eV compared to the other elements of the alloy. Mn content in the CCA NP is lower by 28.0% than the expected value, as described earlier for the Ge-based CCA. However, unlike the Ge-based CCA NPs, no visible core–shell structure was detected around the Al-based NPs, and oxygen intensity was significantly lower in line scan analyses (Figure S4, Supporting Information File 1). This suggests that surface oxidation effects were less pronounced, potentially because of differences in particle formation dynamics or the protective role of other alloying elements.

#### Structural characterization of the CCA NPs

**Figure 5:**
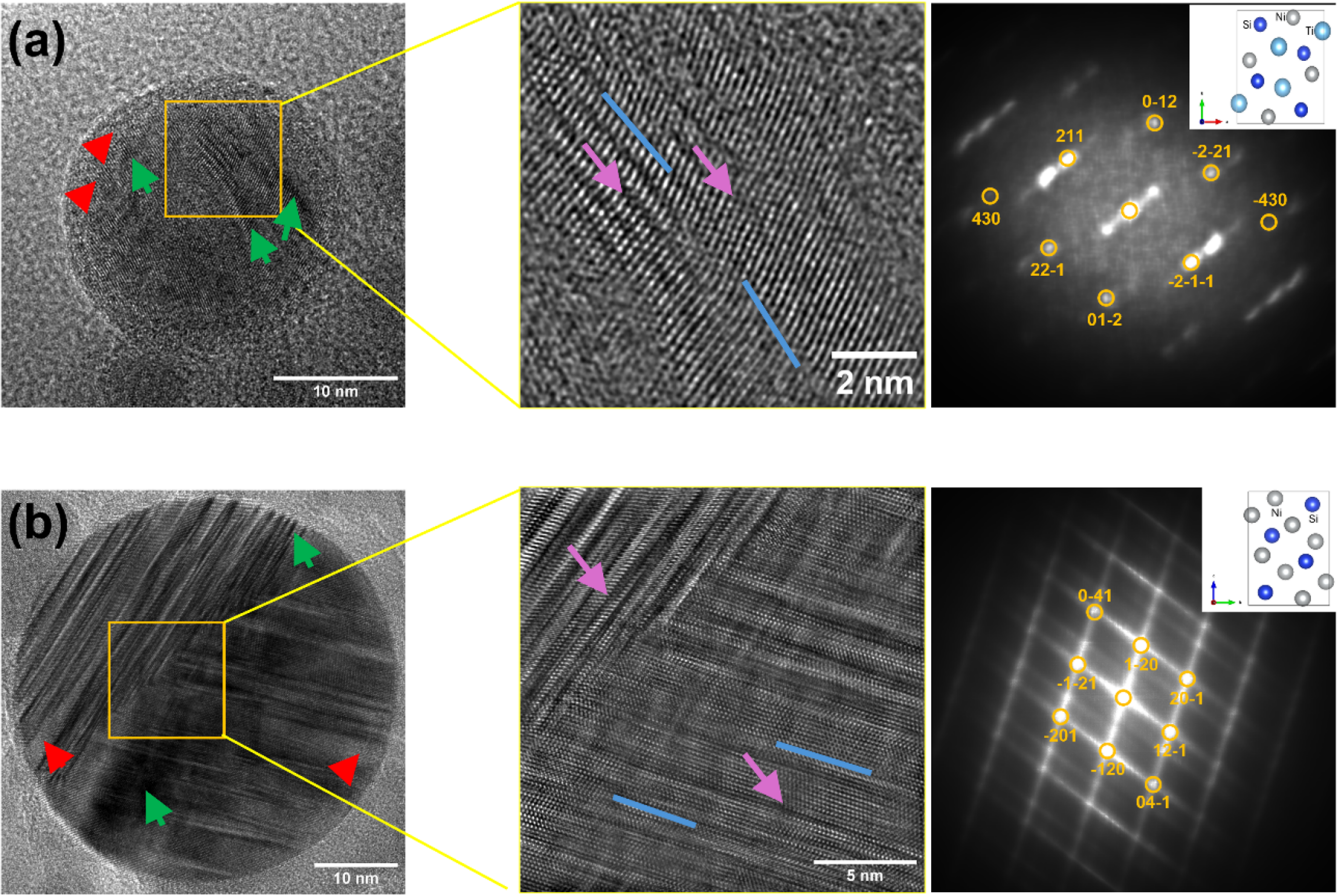
High-resolution TEM images and diffractograms of PLAL-generated CCA NPs. (a) Ge-based CCA NP and (b) Al-based CCA NP.

Figure 5a and Figure 5b show HRTEM bright-field images of the Ge-based CCA NPs and Al-based CCA NPs. The insets reveal the crystallographic structure of the NPs and their corresponding diffractograms. The observed contrast variations (with light and dark areas marked in red and green, respectively) indicate differences in elemental distribution, suggesting the presence of distinct phases and crystalline defects, such as twin boundaries and inhomogeneous stacking of multiple elements (marked with blue and purple arrows, respectively). The line scan of one of the NPs further illustrates deviations in elemental concentration as shown in Figure S3b and Figure S4, Supporting Information File 1. This phenomenon is attributed to the nonequilibrium nature of PLAL, where each laser pulse, occurring on a picosecond timescale, rapidly quenches non-equilibrium phases or highly defective structures [63].

The Ge-based CCA NP diffractogram shows a distribution of diffraction peaks corresponding to an orthorhombic NiSiTi-type crystal structure, with lattice parameters *a* = 0.58492 nm, *b* = 0.70136 nm, and *c* = 0.40149 nm. A previous work by Law et al. using a bulk Ge-based CCA [25] suggested that the orthorhombic crystal structure transforms fully to a hexagonal structure below 190 K. However, the PLAL-synthesized Ge-based CCA NPs do not show a hexagonal structure but remain orthorhombic at room temperature. Additionally, the FCC crystal structure observed in the bulk Ge-based CCA target is not observed.

For the Al-based CCA NPs, the diffractogram reveals numerous bright spots corresponding to an orthorhombic NiSi-type crystal structure with lattice parameters *a* = 0.36062 nm, *b* = 0.51256 nm, and *c* = 0.73241 nm. This aligns with the findings of Biswas et al. [24], where the orthorhombic phase predominates at room temperature for lower Al contents. However, in our XRD analysis of the synthesized bulk Al-based CCA, the orthorhombic crystal structure was not observed. This discrepancy suggests that PLAL influences the resulting crystal structure, paving the way to control the material phase by modifying the laser synthesis conditions such as pulse duration, intensity, or solvent, which would drastically affect temperature, pressure, and cooling rate conditions during NP synthesis [64].

### Magnetic properties of the bulk and nanoscale CCAs

#### Bulk CCAs

**Figure 6:**
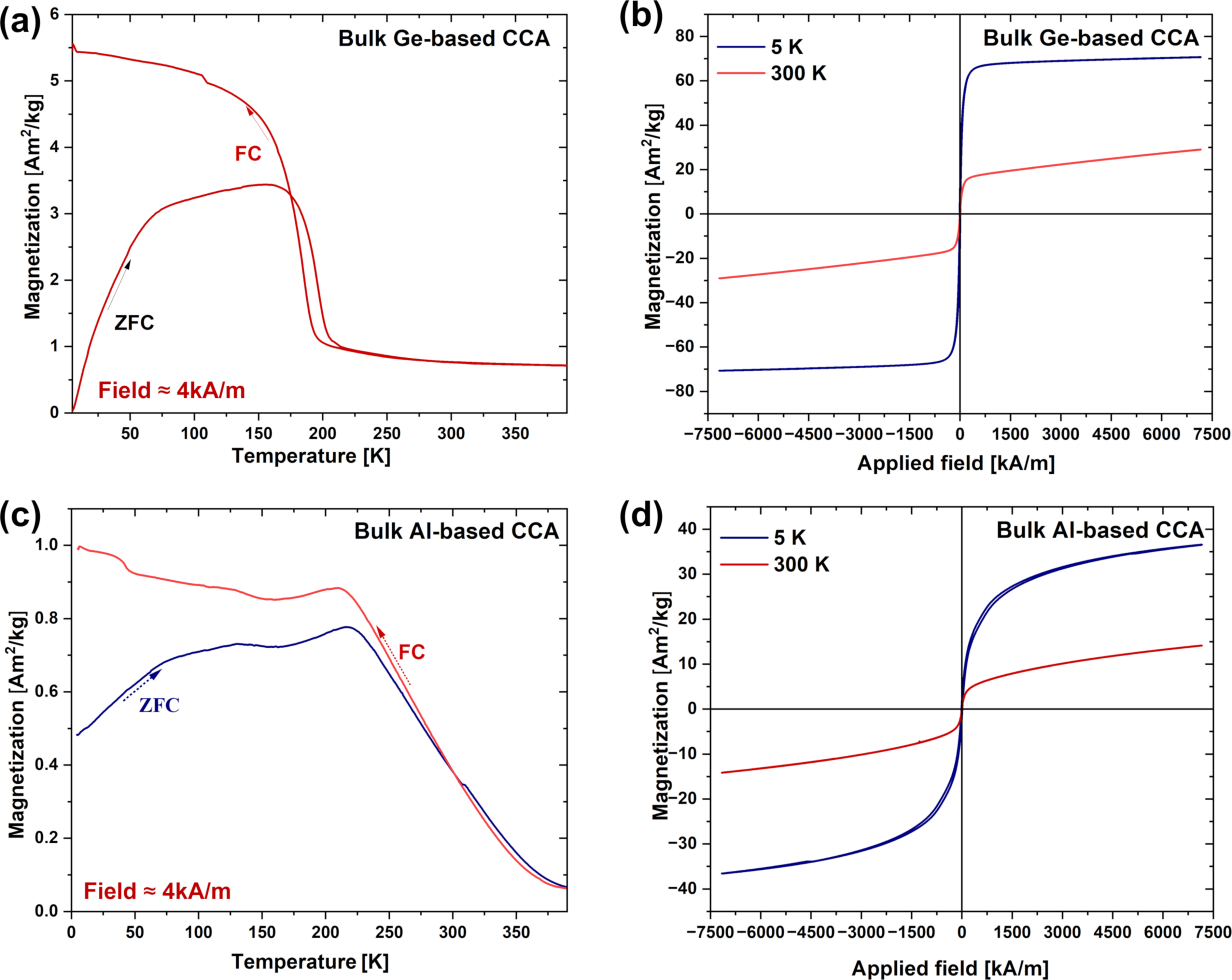
(a) ZFC-FC temperature-dependent magnetization (*M*–*T* curve) of the bulk Ge-based CCA. (b) Field-dependent magnetization curve at 5 and 300 K for the Ge-based CCA. (c) ZFC-FC temperature-dependent magnetization (*M*–*T* curve) of the bulk Al-based CCA. (d) Field-dependent magnetization curve at 5 and 300 K for the Al-based CCA.

The Ge-based bulk CCA shows an abrupt magnetic phase transition, with the zero-field cooled (ZFC) and field cooled (FC) curves starting to diverge around 179 K (Figure 6a), indicating a shift from a paramagnetic to a ferromagnetic state. This transition temperature differs from previous reports that suggested a transition above 170 K. Unlike earlier studies, no metamagnetic transition occurs between 125 and 170 K; instead, the magnetization decreases gradually even under a low field (4kA/m). It is noteworthy that varying the target preparation technique alters the magnetic properties of the material significantly. This is probably related to the low cooling rates employed during SPS (compared to arc melting in a previous study [25]), which result in a different crystal structure and modify the magnetic properties. At temperatures below 179 K, the FC curve shows higher magnetization than the ZFC curve, indicating that spin alignment occurs faster when cooled in the presence of a magnetic field because of the pre-alignment that the magnetic field produces. Above 179 K, the ZFC and FC curves converge, suggesting that thermal energy disrupts magnetic ordering, leading to the appearance of a peak at around 179 K in the d*M*/d*T–T* curve (Figure S5a, Supporting Information File 1), which corresponds to the phase transition temperature, accompanied by a sharp drop in the derivative, indicating a second-order phase transition. This type of transition suggests that the magnetization changes smoothly across the transition point, consistent with ferromagnetic or ferrimagnetic ordering.

The *M*–*H* curve at 5 K (Figure 6b and Figure S5b, Supporting Information File 1) exhibits a pronounced hysteresis loop with a *H*_c_ of 8.4 kA·m^−1^ and a high saturation magnetization *M*_s_ (5 K, 9 T) of 68.2 A·m^2^·kg^−1^, indicating strong ferromagnetic properties (Table 3). At 300 K, the loop becomes narrower, and *H*_c_ drops significantly to 1.2 kA·m^−1^, while *M*_s_ decreases to 18 A·m^2^·kg^−1^. This behavior suggests that the material becomes easier to demagnetize, which is generally good for magnetocaloric cycles. However, the demagnetization comes with a weakened ferromagnetic interaction, likely due to increased thermal agitation at higher temperatures.

**Table 3:** Comparison of magnetic phase transition temperature (*T*_c_), saturation magnetization (*M*_s_), and coercivity (*H*_c_) at 5 and 300 K for bulk Ge-based and Al-based CCAs.

	*T*_c_ [K]	*M*_s_ (5 K) [A·m^2^·kg^−1^]	*M*_s_ (300 K) [A·m^2^·kg^−1^]	*H*_c_ (5 K) [kA·m^−1^]	*H*_c_ (300 K) [kA·m^−1^]

bulk Ge-based CCA	179	68.2	18	8.4	1.2
bulk Al-based CCA	263	32.7	9.5	11	1

The Al-based CCA ZFC and FC curves diverge significantly near 263 K (Figure 6c and Figure S5c, Supporting Information File 1). The higher transition temperature compared to the Ge-based CCA implies stronger magnetic interactions in the Al-based CCA, which are able to persist even at higher temperatures. The broader dip in the d*M*/d*T*–*T* curve, compared to the sharper drop seen in the Ge-based CCA, suggests that the magnetic ordering in the Al-based CCA is more gradual, possibly because of different multiple phases and microstructures. However, the low-temperature magnetization of the Al-based CCA (≈1 A·m^2^·kg^−1^) is much lower than that of the Ge-based CCA (≈5 A·m^2^·kg^−1^), indicating that antiferromagnetic correlations may become important in the Al-based alloy at low temperatures.

The *M*–*H* curves for the Al-based CCA (Figure 6d and Figure S5d, Supporting Information File 1) shows a higher *H*_c_ at 5 K (11 kA·m^−1^) than that observed for the Ge-based CCA (8.4 kA·m^−1^). The *M*_s_ (5 K, 9 T) reaches 32.7 A·m^2^·kg^−1^, which, although significant, is still lower than the 68.2 A·m^2^·kg^−1^ observed for the Ge-based CCA. As the temperature increases to 300 K, the Al-based CCA exhibits a marked decrease in both *H*_c_ (down to 1 kA·m^−1^) and *M*_s_ (9.5 A·m^2^·kg^−1^).

#### CCA NPs

**Figure 7:**
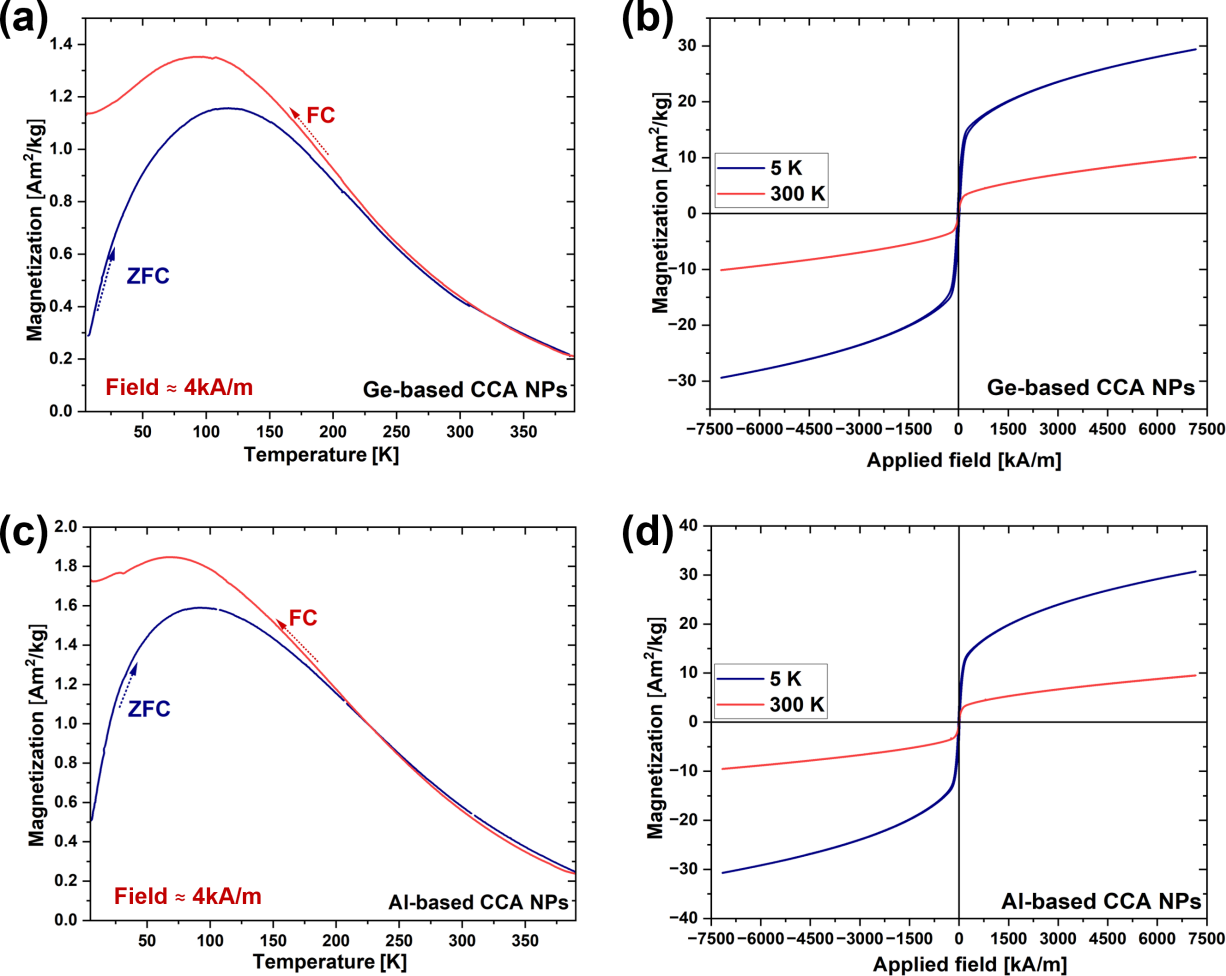
(a) ZFC-FC temperature-dependent magnetization (*M*–*T*) curves of NPs generated from PLAL of the Ge-based CCA target. (b) Field-dependent magnetization curves (*M*–*H*) curves of NPs generated from PLAL of the Ge-based CCA target at 5 and 300 K. (c) ZFC-FC temperature-dependent magnetization (*M*–*T* curve) for NPs generated from PLAL of the Al-based CCA target. (d) Field-dependent magnetization curves (*M*–*H*) curves of NPs generated from PLAL of the Al-based CCA target at 5 and 300 K.

The *M*–*T* curve (Figure 7a) for the Ge-based CCA NPs exhibits a distinct ZFC-FC behavior differing from that of the bulk material. The magnetization increases with temperature, reaching a peak at 100 K, corresponding to the superparamagnetic blocking temperature (*T*_B_). Unlike the bulk CCA, which undergoes an apparent magnetic phase transition at *T*_c_ = 179 K, the NPs experience a superparamagnetic-to-paramagnetic transition at 204 K (Figure S6a, Supporting Information File 1).

The magnetization *M* (5 K, 50 mT) for the NPs (≈1.2 A·m^2^·kg^−1^) is lower than that of the bulk (≈5 A·m^2^·kg^−1^) mainly because of the blocking temperature effect. Below *T*_B_, the thermal energy is sufficient to cause flipping of magnetic moments, reducing the measured magnetization at low fields compared to the bulk, where the moments remain fully aligned. Additionally, the formation of an oxide shell (≈2.5 nm thick) on Ge-based NPs further contributes to a decrease in *M*_s_ by reducing the magnetically active volume.

At 5 K, the *M*–*H* curve displays a significant hysteresis loop with a *H*_c_ of 26.2 kA·m^−1^, which is higher than that of the bulk Ge-based CCA (8.4 kA·m^−1^) (Figure 7b, Table 4, and Figure S6b, Supporting Information File 1). At 300 K, *H*_c_ drops to 0.7 kA·m^−1^, and *M*_s_ (300 K, 9 T) decreases to 6.6 A·m^2^·kg^−1^, indicating that the thermal energy at room temperature is sufficient to overcome the magnetic anisotropy. The value of *M*_s_ (5 K, 9 T) for the bulk material (68.2 A·m^2^·kg^−1^) is much higher than that of the NPs (25.1 A·m^2^·kg^−1^). This reduction in *M*_s_ in the NPs can be attributed to the inhomogeneity of the NPs and the formation of oxide shells. At 300 K, *H*_c_ drops to 0.7 kA·m^−1^, and *M*_s_ decreases to 6.6 A·m^2^·kg^−1^, indicating that the thermal energy at room temperature is sufficient to overcome the magnetic anisotropy, leading to a weaker magnetic response. The larger drop in *M*_s_ and *H*_c_ in the NPs with increasing temperature reflects the greater impact of thermal fluctuations on nanoscale materials.

The magnetic response of the Al-based CCA NPs exhibited a behavior (Figure 7c,d) similar to that of the Ge-based CCA NPs. The magnetization increases with temperature, reaching a peak at superparamagnetic blocking temperature of around 100 K and then decreases, indicating a superparamagnetic-to-paramagnetic transition. A divergence between ZFC and FC curves is observed below 202 K (Figure S6c, Supporting Information File 1), suggesting spin freezing or magnetic domain alignment when the material is cooled in the presence of a magnetic field.

The *M*–*H* curve (Figure 7d and Figure S6d, Supporting Information File 1) of the Al-based CCA at 5 K shows significant hysteresis, with a *H*_c_ of 16.6 kA·m^−1^, which is higher than that of the bulk Al-based CCA (11 kA·m^−1^). The increase in *H*_c_ in the NPs is likely due to enhanced surface anisotropy effects that arise from the smaller particle size. However, *M*_s_ (300 K, 9 T) and *M*_s_ (5 K, 9 T) of the Al-based CCA NPs are comparable, while a lower *H*_c_ is exhibited at low temperature (5 K) compared to Ge-based CCA NPs (Table 4). This decrease in *H*_c_ can be attributed to absence of oxide shell formation and the difference in composition and crystal structure.

**Table 4:** Comparison of saturation magnetization (*M*_s_) and coercivity (*H*_c_) at 5 and 300 K for Ge-based and Al-based CCAs NPs.

	*M*_s_ (5 K) [A·m^2^·kg^−1^]	*M*_s_ (300 K) [A·m^2^·kg^−1^]	*H*_c_ (5 K) [kA·m^−1^]	*H*_c_ (300 K) [kA·m^−1^]

Ge-based CCA NPs	25.1	6.6	26.2	0.7
Al-based CCA NPs	25.9	6.8	16.6	1.1

## Conclusion

We present rare-earth-free Ge-based and Al-based CCAs as promising candidates for low-temperature magnetic applications. In the bulk state, the Ge-based CCA exhibits a *T*_c_ of 179 K, while the Al-based CCA has a higher *T*_c_ of 263 K. The Ge-based CCA exhibits almost twice the *M*_s_ and a ≈20% lower *H*_c_ than the Al-based CCA at 5 and 300 K.

At the nanoscale, both CCAs exhibit superparamagnetic behavior with *T*_B_ of 120 K for Ge-based NPs (*x*_c_ = 13.4 ± 9.1 nm) and 100 K for Al-based NPs (*x*_c_ = 18.4 ± 15.1 nm). While the Ge-based NPs maintain a higher *M*_s_, the Al-based NPs exhibit a comparable *M*_s_ and an approximately 45% lower *H*_c_ at low temperature (5 K). This reduction in coercivity makes the Al-based CCA NPs particularly attractive for applications requiring soft magnetic materials with enhanced magnetocaloric performance and lower hysteretic losses at low temperatures.

Beyond their magnetic properties, Al-based CCAs offer notable economic and material availability advantages. As Al is significantly more cost-effective and abundant than Ge, Al-based CCAs present a scalable and sustainable alternative for rare-earth-free magnetic materials. This substitution is particularly relevant for future advancements in magnetocaloric refrigeration, sensors, and other energy-efficient technologies.

## Supporting Information

File 1 Additional experimental data.

## Data Availability

Data generated and analyzed during this study is available from the corresponding author upon reasonable request.
