# Supplementary material for: Synthesis and magnetic transitions of rare-earth-free Fe–Mn–Ni–Si-based compositionally complex alloys at bulk and nanoscale
Source: Beilstein J Nanotechnol. 2025 Jun 5;16:823–36. doi: 10.3762/bjnano.16.62 (PMC12152319; doi:10.3762/bjnano.16.62)
Supplement: File 1 — Additional experimental data. [file Beilstein_J_Nanotechnol-16-823-s001.pdf]

BEILSTEIN JOURNAL OF NANOTECHNOLOGY

Supporting Information

for

Synthesis and magnetic transitions of rare-earth-free Fe–Mn–Ni–Si-based compositionally complex alloys at bulk and nanoscale

Shabbir Tahir, Tatiana Smoliarova, Carlos Doñate-Buendía, Michael Farle, Natalia Shkodich and Bilal Gökce

Beilstein J. Nanotechnol. **2025,** *16,* 823–836. doi:10.3762/bjnano.16.62

Additional experimental data

License and Terms: This is a supporting information file under the terms of the Creative Commons Attribution License (https://creativecommons.org/licenses/by/4.0). Please note that the reuse, redistribution and reproduction in particular requires that the author(s) and source are credited and that individual graphics may be subject to special legal provisions.
The license is subject to the *Beilstein Journal of Nanotechnology* terms and conditions: (https://www.beilstein-journals.org/bjnano/terms)

1. Elemental composition of Bulk Ge-based CCAs

The ground and polished surface were re-examined, along with the cross-section of the bulk Ge-based CCA sample using SEM/EDX. Figures S1a and S1b present the elemental distribution maps obtained from the surface and cross-section, respectively. The results indicate a highly homogeneous distribution of Mn, Fe, Ni, Ge, and Si across both the surface and the interior of the alloy. The quantitative EDX data (Table S1) further confirm that the average elemental composition closely matches the intended bulk composition, with only minor variations observed between different regions. These findings, in agreement with the results shown in the main manuscript, demonstrate that the surface and the internal volume of the alloy are compositionally consistent within experimental error.

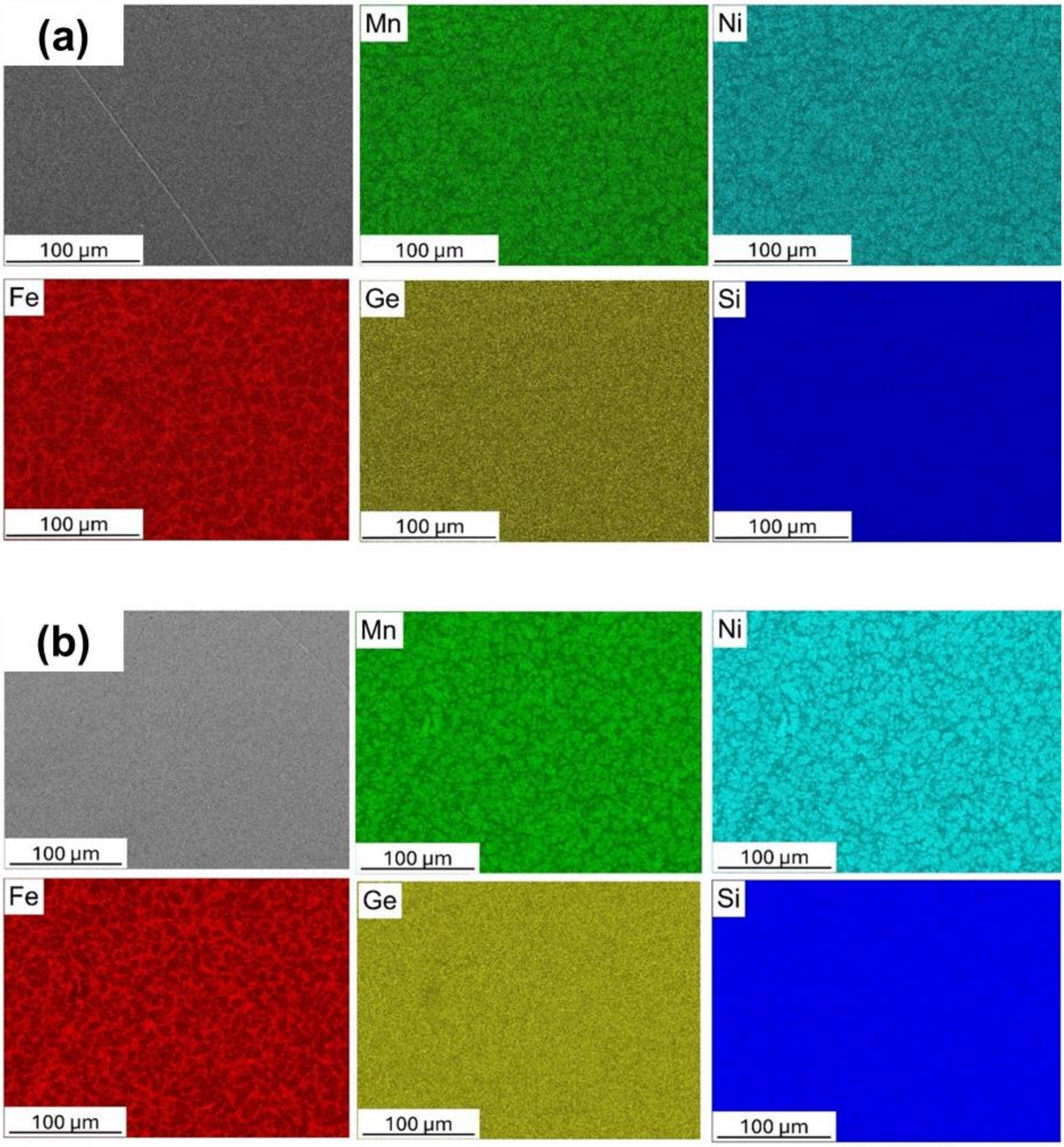

Figure S1: (a): SEM (SE) and EDX elemental maps of Mn, Fe, Ni, Ge and Si obtained from the surface of the bulk Ge-based CCA. (b): SEM (SE) and EDX elemental maps of Mn, Fe, Ni, Ge and Si obtained from the cross-section of the bulk Ge-based CCA.

Table S1: EDX results performed from the fine-polished surface, and the cross-section of the Bulk-Ge based CCA sample.

Bulk Ge-based CCA	Mn [atom %]	Ni [atom %]	Fe [atom %]	Ge [atom %]	Si [atom %]
Surface	21.66 ± 0.06	20.95 ± 0.04	23.91 ± 0.08	15.76 ± 0.06	17.71 ± 0.05
Cross-section	21.57 ± 0.08	20.91 ± 0.06	23.98 ± 0.08	15.92 ± 0.04	17.63 ± 0.07

2. Local elemental composition of Bulk Al-based CCAs.

Figure S2 and Table S2 below present the local compositions of the bulk Al-based CCA, measured at various positions. The table highlights the formation of multiphase alloying or possible alloying inconsistencies, with noticeable segregation of Al and Mn in most cases.

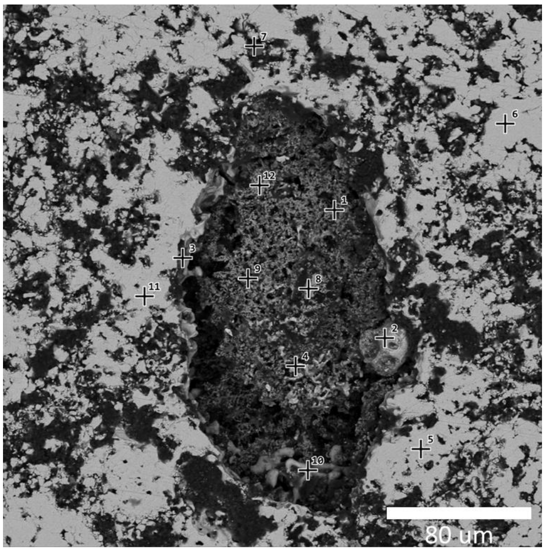

Figure S2: SEM image of bulk Al-based CCA, with marked positions indicating where EDX mapping was performed.

Table S2: Elemental composition by SEM EDX-mapping at local position of bulk Al-based CCA.

	Ni (atom %)	Mn (atom %)	Fe (atom %)	Si (atom %)	Al (atom %)
1	31.94	11.42	20.54	36.09	-
2	32.56	11.36	20.55	35.52	-
3	34.72	30.62	-	18.23	16.43
4	34.72	30.62	-	18.23	16.43
6	11.61	58.79	12.12	17.49	-
7	28.49	23.49	46.74	1.25	-
8	38.32	15.07	38.91	4.23	2.97
9	-	89.01	-	10.99	-
10	-	78.19	-	21.81	-
11	12.6	29.74	50.11	4.84	2.69
12	35.09	30.40	8.08	26.41	-

3. Formation of core–shell structure for Ge-based CCA NPs

Figure S3 presents the HRTEM image and EDX line scan of a Ge-based CCA NP. The HRTEM image clearly shows the formation of an amorphous shell around the NP, indicating surface oxidation. The EDX line scan confirms the presence of all constituent elements within the NP, while a higher oxygen intensity at the edges suggests the formation of an oxide layer on the surface. Since oxidation is predominantly observed at the surface, it likely occurred after the PLAL process, possibly during nanoparticle colloid deposition and drying on the TEM grid or due to interactions with the solvent.

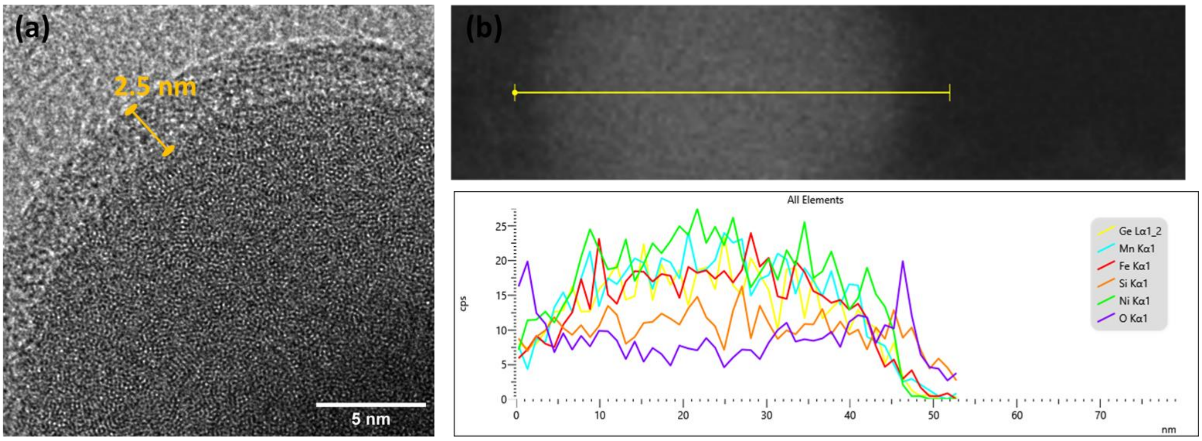

Figure S3: (a) HRTEM image and (b) EDX line scan of a Ge-based CCA NP, showing the elemental distribution of Ni, Mn, Fe, Si, Ge, and O. The higher oxygen intensity at the NP's surface indicates the formation of an oxide layer.

4. EDX line scan of Al-based CCA NP

The TEM image of Al-based CCA confirm that no shell formation was observed in Al-based NPs and the EDX line scan reveals that all constituent elements (Ni, Mn, Fe, Al and Si) were present. The O peak was same throughout, showing that some oxide phases could be formed within the particle.

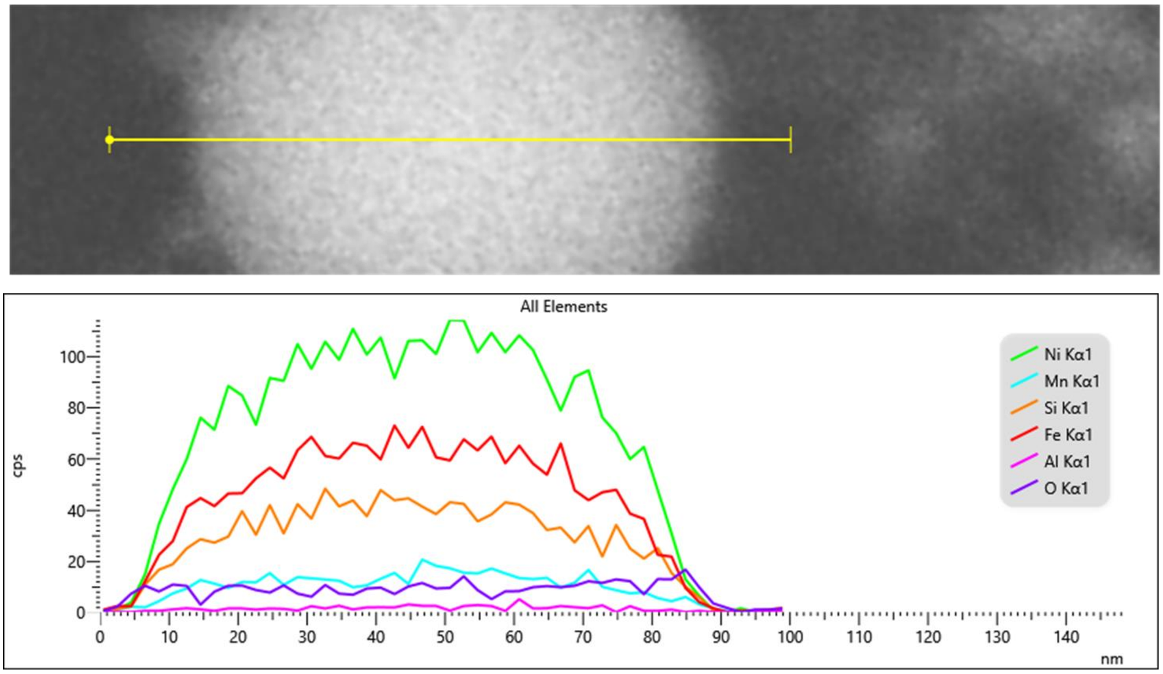

Figure S4: TEM image and EDX line scan of an Al-based CCA NP, showing the elemental distribution of Ni, Mn, Fe, Si, Al, and O.

5. Magnetic properties of bulk CCA (Additional information)

Figure S5a shows the temperature derivative of magnetization (d*M*/d*T*) obtained from ZFC-FC *M*–*T* curves of the bulk Ge-based CCA measured at an applied field of ≈4kA/m. A sharp minimum is observed at 179 K, indicating a well-defined second-order ferromagnetic-to-paramagnetic phase transition. The field-dependent magnetization (*M*–*H*) curves of the bulk Ge-based CCA are shown near zero field for 5 K (blue) and 300 K (red) in Figure S5b. At 5 K, the loop exhibits clear hysteresis, with coercivity of H_c = 8.4 kA/m while at 300 K the coercivity decreases to H_c = 1.2 kA/m). The d*M*/d*T* curve (Figure S5c) for the bulk Al-based CCA displays a broader and shallower minimum at around 263 K, corresponding to its Curie temperature. The broader transition suggests either multiphase behaviour or more gradual magnetic ordering compared to the Ge-based alloy. The *M*–*H* curves of the Al-based CCA (Figure S5c) show H_c = 11.0 kA/m at 5 K and 1.0 kA/m at 300 K.

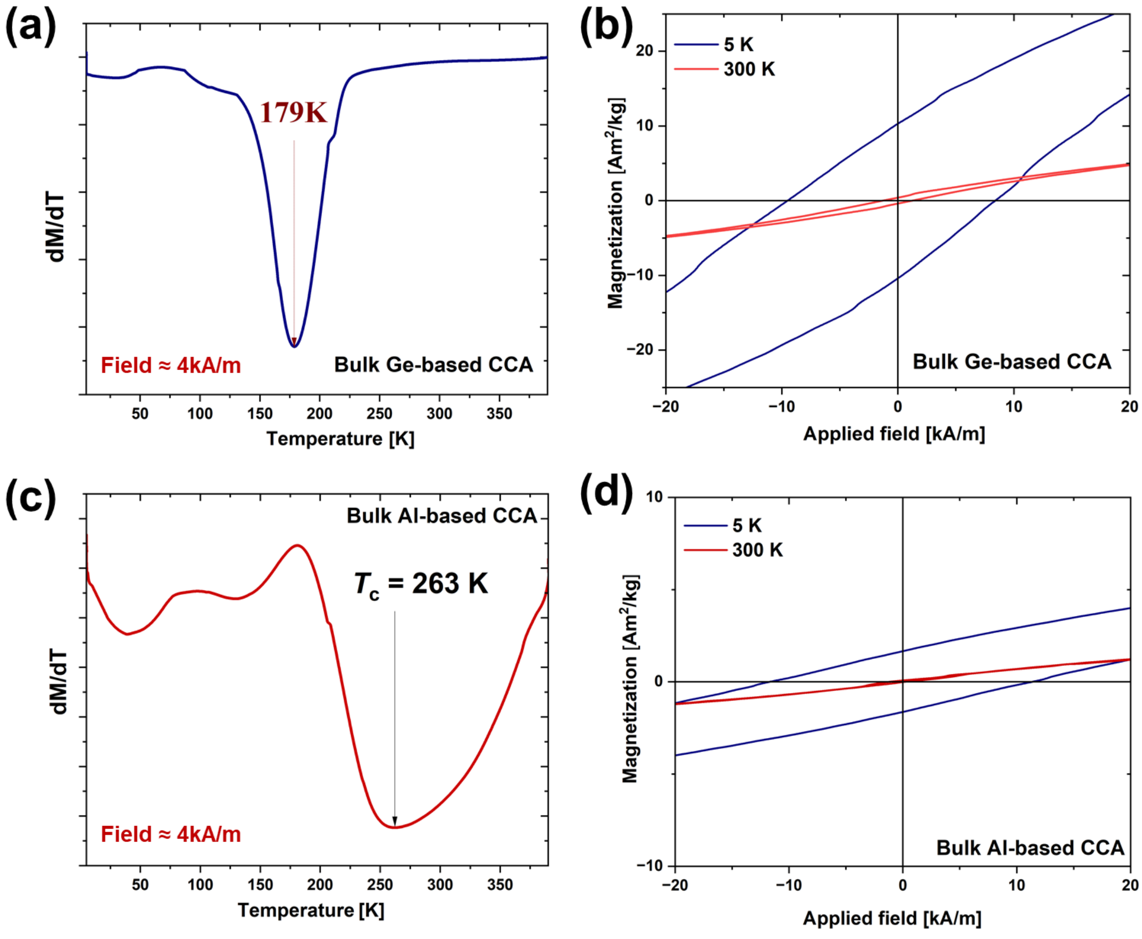

Figure S5: (a) the magnetization derivative with the temperature of ZFC-FC M-T curve of bulk Ge-based CCA. (b) Field-dependent magnetization near 0 T at 5 K (blue) and 300 K (red) for bulk Ge-based CCA. (c) The magnetization derivative with the temperature of ZFC-FC *M*–*T* curve of bulk Al-based CCA. (d) Field-dependent magnetization near 0 T at 5 K (blue) and 300 K (red) for bulk Al-based CCA.

6. Magnetic properties of CCA NPs (Additional information)

The d*M*/d*T* for Ge-based CCA nanoparticles, measured at ≈4kA/m (Figure S6a), shows a magnetic transition at 204 K, indicating a shift from superparamagnetic to paramagnetic behavior. The *M*–*H* curves of Ge-based CCA NPs at 5 K (blue) and 300 K (red) show significant hysteresis at low temperature, with H_c = 26.2 kA/m, and a nearly linear response at 300 K, with H_c = 0.7 kA/m. The d*M*/d*T* curve for Al-based CCA NPs shows a magnetic transition at 202 K (Figure S6c), similar to the Ge-based

system. The *M–H* curves of Al-based CCA NPs show H_c = 16.6 kA/m at 5 K and 1.1 kA/m at 300 K. These results show that both nanoparticle systems exhibit superparamagnetic-to-paramagnetic transitions and Al-based NPs show lower coercivity at low temperatures.

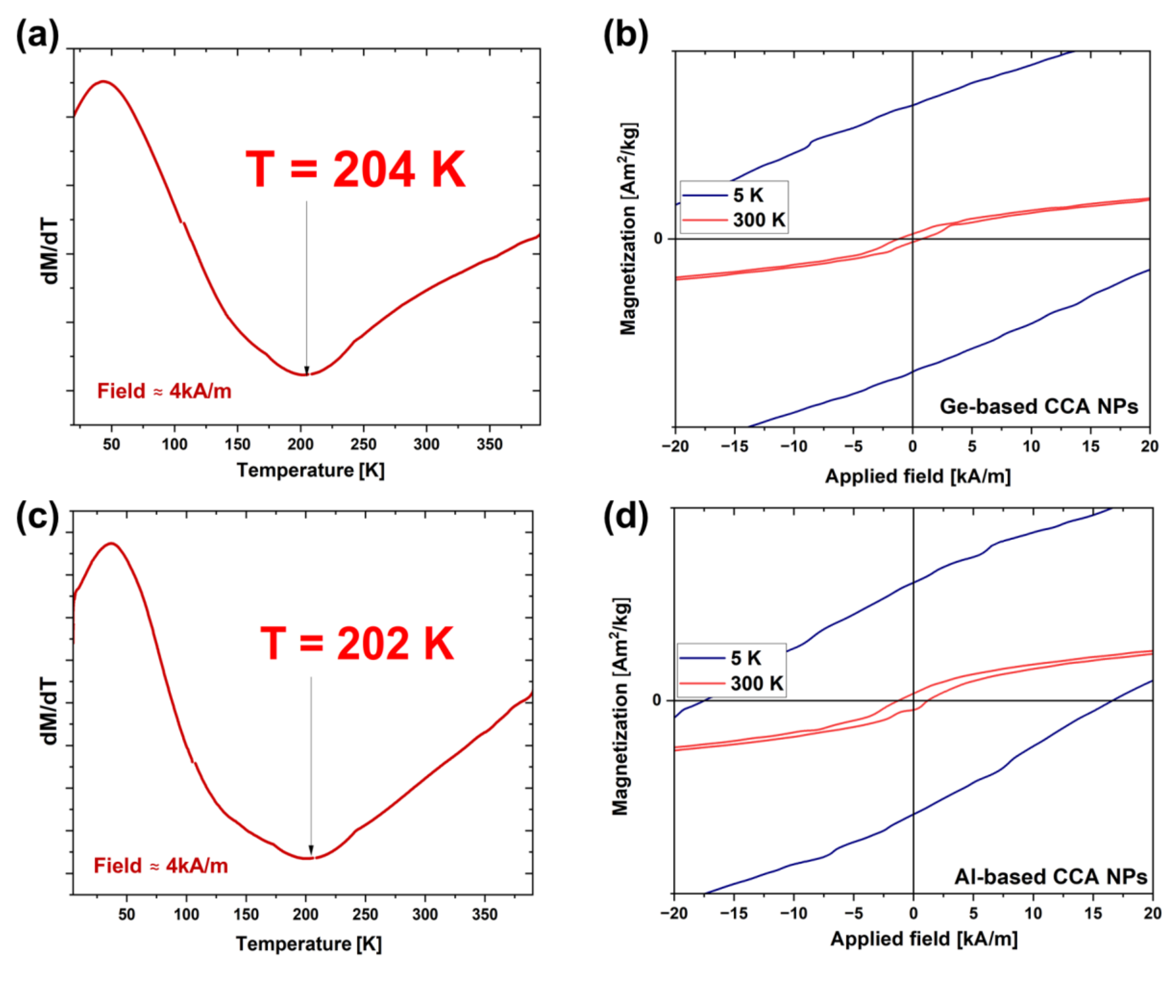

Figure S6: (a) The magnetization derivative with the temperature of ZFC-FC *M–T* curve of Ge-based CCA NPs. (b) Field-dependent magnetization near 0 T at 5 K (blue) and 300 K (red) and of Ge-based CCA NPs. (c) The magnetization derivative with the temperature of ZFC-FC *M–T* curve of Al-based CCA NPs. (d) Field-dependent magnetization near 0 T at 5 K (blue) and 300 K (red) for Al-based CCA NPs.
